# Phenotypic and genotypic characteristics associated with biofilm formation in clinical isolates of atypical enteropathogenic *Escherichia coli* (aEPEC) strains

**DOI:** 10.1186/1471-2180-14-184

**Published:** 2014-07-10

**Authors:** Heloisa H Nascimento, Lucas EP Silva, Renata T Souza, Neusa P Silva, Isabel CA Scaletsky

**Affiliations:** 1 Departamento de Microbiologia, Imunologia e Parasitologia, Universidade Federal de São Paulo, Rua Botucatu, 862, 3 andar, 04023-062 São Paulo, Brazil; 2 Disciplina de Reumatologia, Universidade Federal de São Paulo, São Paulo, Brazil

**Keywords:** Enteropathogenic *Escherichia coli*, Biofilm formation, Type 1 pili

## Abstract

**Background:**

Biofilm formation by enteropathogenic *Escherichia coli* (EPEC) have been recently described in the prototype typical EPEC E2348/69 strain and in an atypical EPEC O55:H7 strain. In this study, we sought to evaluate biofilm formation in a collection of 126 atypical EPEC strains isolated from 92 diarrheic and 34 nondiarrheic children, belonging to different serotypes. The association of biofilm formation and adhesin-related genes were also investigated.

**Results:**

Biofilm formation occurred in 37 (29%) strains of different serotypes, when the assays were performed at 26°C and 37°C for 24 h. Among these, four strains (A79, A87, A88, and A111) formed a stronger biofilm than did the others. The frequency of biofilm producers was higher among isolates from patients compared with isolates from controls (34.8% vs 14.7%; P = 0.029). An association was found between biofilm formation and expression of type 1 fimbriae and curli (P < 0.05). Unlike the previously described aEPEC O55:H7, one aEPEC O119:HND strain (A111) formed a strong biofilm and pellicle at the air-liquid interface, but did not express curli. Transposon mutagenesis was used to identify biofilm-deficient mutants. Transposon insertion sequences of six mutants revealed similarity with type 1 fimbriae (*fimC*, *fimD*, and *fimH*), diguanylate cyclase, ATP synthase F1, beta subunit (atpD), and the uncharacterized YjiC protein. All these mutants were deficient in biofilm formation ability.

**Conclusion:**

This study showed that the ability to adhere to abiotic surfaces and form biofilm is present in an array of aEPEC strains. Moreover, it seems that the ability to form biofilms is associated with the presence of type 1 fimbriae and diguanylate cyclase. Characterization of additional biofilm formation mutants may reveal other mechanisms involved in biofilm formation and bring new insights into aEPEC adhesion and pathogenesis.

## Background

Enteropathogenic *Escherichia coli* (EPEC) is an important cause of infantile diarrhea, particularly in developing countries [1]. EPEC causes protracted and chronic diarrhea, and the severity of this disease may require extensive hospitalization [2-4]. The majority of EPEC isolates belong to classic serotypes derived from 12 classical O serogroups (O26, O55, O86, O111, O114, O119, O125, O126, O127, O128, O142, and O158) [5,6]. A key characteristic of EPEC strains is the ability to intimately attach to intestinal epithelial cells and form attaching and effacing (AE) lesions. These lesions are characterized by the destruction of the microvilli and the rearrangement of the cytoskeleton, culminating in a pedestal-like structure at the site of bacterial contact [7]. The AE genes are localized to the locus for enterocyte effacement (LEE) and encode intimin, a type III secretion system, secreted proteins (Esp) and the translocated intimin receptor [8-10]. Another EPEC characteristic is the formation of microcolonies on cell monolayers “in vitro”, a pattern known as localized adherence (LA) [11]. EPEC forms microcolonies on cultured epithelial cells, cultures of pediatric small intestinal tissue, and biopsy samples from EPEC patients [12,13].

“Typical” EPEC (tEPEC) also contains the EPEC adherence factor (EAF) plasmid [14], which carries genes encoding a regulator (*per*) [15] and the bundle-forming pili (BFP) [16] that mediates LA to epithelial cells. EPEC strains, lacking the EAF plasmid are deprived of BFP, and have been designated “atypical” EPEC (aEPEC) [17]. aEPEC strains can still adhere to HEp-2 cells in a localized adherence-like pattern (LAL), forming loose bacterial microcolonies [18]. Recent epidemiological studies indicate that aEPEC is more prevalent than tEPEC in both developed and developing countries [1].

The aEPEC strains are genetically related to the enterohemorrhagic *E. coli* (EHEC), and both are considered emerging pathogens [19]. Some previous studies showed that certain EHEC strains have the abilities to attach, colonize, and form biofilm on various surfaces [20-24]. Some adhesins such as type 1 pilus (T1P), flagella, HCP (“hemorrhagic coli pilus”), curli, antigen 43 (Ag43), calcium-binding antigen 43 homologue (Cah), and autotransporter protein of EHEC (EhaA) have been implicated in the formation of microcolonies and biofilms [20-24]. Even though EPEC form microcolonies and causes persistent infections, very little is known regarding biofilm formation by EPEC strains, and their survival outside the host.

Recently, Moreira et al. [25] published the first description of biofilm formation by the prototype tEPEC E2348/69 (O127:H6) strain. These researchers found that adhesive structures, such as type 1 fimbriae, Ag43, BFP, and EspA, were expressed during the initial stages of biofilm development. More recently, Weiss-Muszkat et al. [26] reported a multicellular behavior characterized by the formation of a robust biofilm on an abiotic surface at 26°C, but not at 37°C, a dense pellicle at the air-liquid interface and a red, dry, and rough (rdar) morphotype in one O55:H7 aEPEC strain. Transposon mutagenesis analysis identified curli fibers and the Crl regulator as important participants in the formation of all three types of biofilms. The aim of this study was to evaluate the capacity of biofilm formation in a collection of 126 aEPEC strains isolated from diarrheic and nondiarrhiec children. The presence of adhesins related to biofilm formation and the association of biofilm formation and these adhesins were also investigated.

## Results and discussion

**Figure 1:**
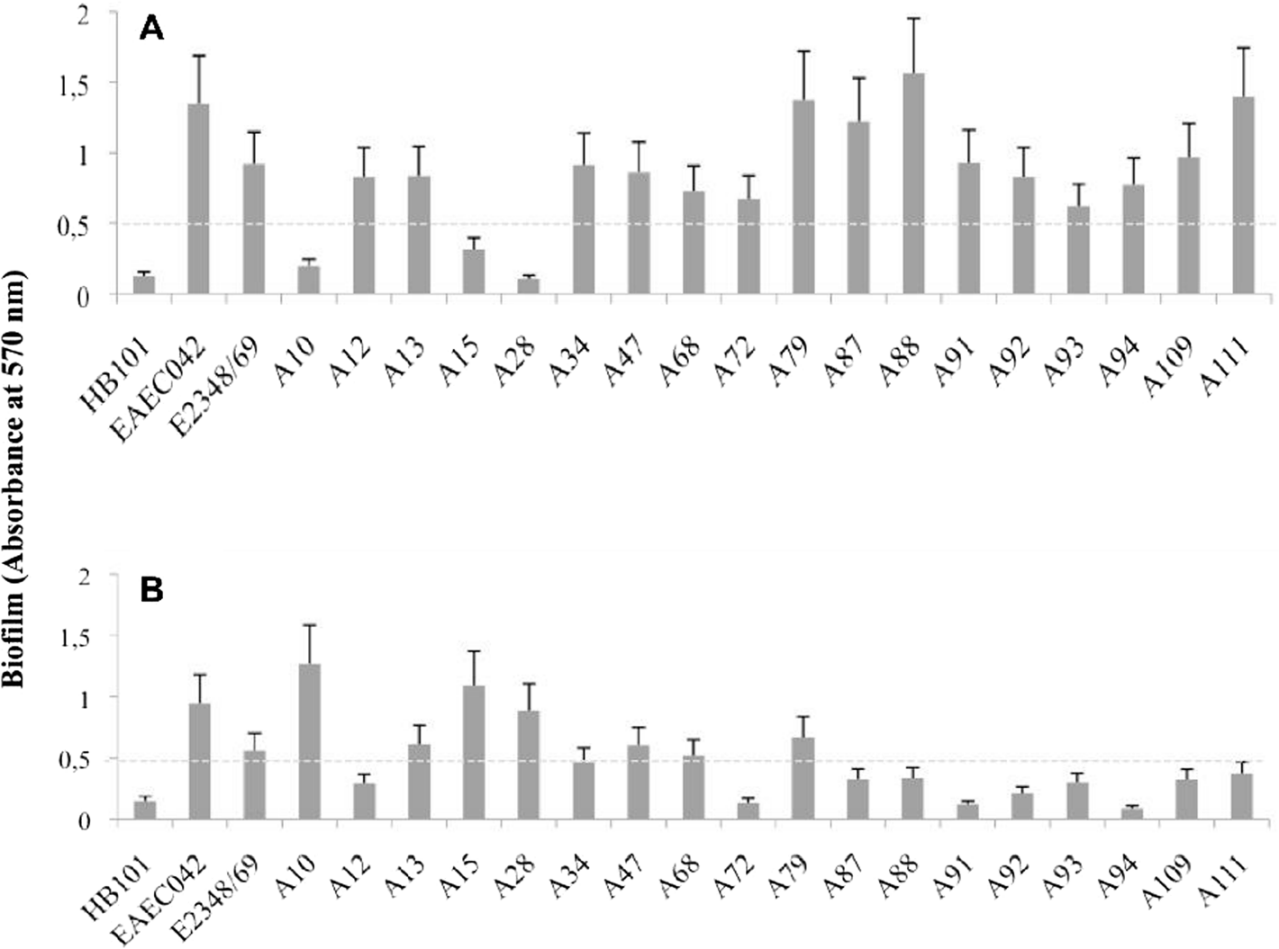
**Biofilm formation on 96-well polystyrene microtiter plates by representative biofilm-producing aEPEC strains.** Strains were incubated in LB broth for 24 h at 26°C **(A)** and 37°C **(B)**, and classified as biofilm producers when OD_570_ was higher than the *E. coli* HB101 strain mean value (OD_570_ ≤ 0.5). Data are from a representative experiment with three replicates. The bars represent the standard deviation.

A total of 126 EPEC strains isolated from 92 children with diarrhea and 34 asymptomatic controls were examined for the capacity of biofilm formation. As shown in Table 1, biofilm formation occurred in 37 (29.4%) of strains of different serotypes after 24 h of incubation at 26°C and 37°C. The frequency of biofilm producers was higher among isolates from patients compared to isolates from controls (34.8% vs 14.7%; P = 0.029). Most of the biofilm-producing strains formed biofilms on an abiotic surface at 26°C (Figure 1A), but not at 37°C (Figure 1B). Among these, four strains (A79, A87, A88, and A111) formed a stronger biofilm than did the others.

**Table 1:** Biofilm formation in 126 clinical isolates of aEPEC strains

**Serotype**		**No. of strains (total/biofilm producers)**^**a**^		
		**Patients**	**Controls**	**Total**
EPEC serotypes				
	O26:H11;HND	9/2	1/0	10/2
	O55:HND	4/1	1/0	5/1
	O111:NM	2/1	2/1	4/2
	O114:NM	0	1/0	1/0
	O119:H2;HND	9/4	1/0	10/4
	O125:HND	0	1/0	1/0
	O126:NM	1/1	0	1/1
	O127:NM;H40	4/2	1/0	5/2
	O128:NM	2/0	0	2/0
	O142:NM;H2	10/1	0	10/1
Non-EPEC serotypes^b^		23/5	12/2	35/7
ONT:H18/NM; HND		28/15	14/2	42/17
Total		92/32	34/5^c^	126/37

^a^Biofilm assays were performed in 96-well polystyrene microtiter plates in Luria broth and incubated for 24 h at 26°C and 37°C.

^b^O4:HND; O15:HND O33:H6; O35:H19; O37:HND; O49:HND; O61:HND; O63:HND; O79:HND; O85:H40; O96:HND; O98:HND; O101:HNM; O103:HNM; O105:H7; O108:H31; O109:H54; O117:HND; 0132:HND; O141:HND; O1523H2; O156:H16; O157:NM; O167:H6; O169:H6; O175:HND.

^C^P < 0.05 (patients x controls).

As shown in Table 2, the *fimA*/*fimH* genes were identified in 27 (73%) strains, and the expression of type 1 fimbriae, was identified in all of them by MSHA assays. The genes related to curli (*csgA* and *crl*) were identified in 17 (45.9%) strains, and all of them expressed curli as judged by characteristic red-colonies formed on CR agar plates. In addition, 18 of strains formed a thick pellicle at the air-liquid interface at 26°C and 37°C. The flagella gene (*fliC)* was identified in 11 (29.8%) strains. The genes related to the autotransporter proteins Ag43 (*flu*), Cah (*cah*), and EhaA (*ehaA*) occurred in 9 (24.3%), 7 (18.9%), 5 (13.5%) strains, respectively. The gene *hcpA* was identified in two strains.

**Table 2:** Phenotypic and genotypic characteristics of aEPEC biofilm-producing strains

**Strain**	**Serotype**	**Source**	**Biofilm formation**				**Expression of adhesins**			**Adhesin gene sequences**						
			**Abiotic surface**		**Air-liquid interface**		**Curli**		**T1F**^**a**^							
			**26°C**	**37°C**	**26°C**	**37°C**	**26°C**	**37°C**		***fimA***	***fliC***	***csgA***	***hcpA***	***flu***	***cah***	***ehaA***
A91	O15:HND	Patient	+	-	+	-	+	+	+	+	-	+	-	-	-	-
A72	O26:HND	Patient	+	-	+	-	-	+	-	-	+	+	-	+	-	-
A132	O26:HND	Patient	+	-	+	-	-	-	+	+	+	-	-	+	-	-
A113	O55:HND	Patient	+	-	+	-	-	-	+	+	+	-	+	-	-	-
A15	O85:H40	Patient	-	+	-	-	-	+	+	+	-	+	-	-	+	+
A28	O103:NM	Patient	-	+	-	-	-	+	+	+	+	+	-	-	+	-
A4	O111:NM	Control	-	+	-	-	-	+	+	+	-	+	-	-	-	+
A12	O111:NM	Patient	+	-	-	+	-	-	+	+	-	-	-	+	-	-
A60	O119:H2	Patient	+	+	-	+	-	-	+	+	-	-	-	-	-	-
A67	O119:H2	Patient	-	+	-	+	-	-	+	+	-	-	-	-	-	-
A93	O119:HND	Patient	+	-	+	+	-	-	+	+	-	-	-	+	-	-
A111	O119:HND	Patient	+	-	+	+	-	-	+	+	-	-	-	-	-	-
A13	O126:NM	Patient	+	+	-	-	-	-	+	+	-	-	-	+	-	-
A5	O127:H40	Patient	+	+	+	-	+	-	-	-	-	+	-	-	-	-
A34	O127:NM	Patient	+	-	-	-	-	-	-	-	+	-	-	-	-	+
A47	O141:HND	Control	+	+	-	-	-	+	-	-	-	+	-	-	+	-
A11	O142:NM	Patient	+	+	-	-	-	-	-	-	-	-	-	+	-	-
A87	O153:H2	Patient	+	-	+	+	+	+	+	+	+	+	-	-	-	-
A36	O157:HND	Control	+	+	+	-	-	-	-	-	-	-	-	-	+	-
A92	O167:H6	Patient	+	-	+	-	+	-	+	+	-	+	-	-	-	-
A10	ONT:HND	Patient	-	+	-	-	-	-	+	+	-	-	-	+	-	-
A20	ONT:HND	Patient	+	+	-	-	-	+	+	+	-	+	-	-	-	-
A22	ONT:HND	Patient	-	+	-	-	-	-	+	+	-	-	-	-	-	-
A39	ONT:NM	Patient	+	+	-	-	-	+	+	+	-	+	-	-	-	-
A48	ONT:HND	Control	+	-	-	-	-	+	-	-	-	+	-	-	+	-
A49	ONT:HND	Patient	-	+	+	-	+	+	-	-	-	+	-	-	-	-
A68	ONT:HND	Patient	+	+	+	-	+	+	+	+	-	+	-	-	+	-
A73	ONT:HND	Control	+	-	-	-	-	-	+	+	+	-	-	-	-	-
A79	ONT:HND	Patient	+	+	+	-	-	-	+	+	+	-	-	-	-	-
A88	ONT:HND	Patient	+	-	+	+	-	-	+	+	-	-	-	-	-	-
A94	ONT:HND	Patient	+	-	-	-	-	-	-	-	-	-	-	-	+	-
A98	ONT:HND	Patient	+	-	-	-	-	+	+	+	+	+	-	-	-	+
A99	ONT:HND	Patient	+	+	-	+	-	-	+	+	+	-	-	-	-	+
A112	ONT:HND	Patient	+	+	+	-	-	-	+	+	-	-	-	-	-	-
A146	ONT:HND	Patient	+	+	+	+	+	+	+	+	-	+	-	+	-	-
A109	ONT:HND	Patient	+	-	+	+	+	-	-	-	-	+	-	-	-	-
A157	ONT:HND	Patient	+	+	+	+	-	-	+	+	+	-	+	+	-	-
Total no. of strains (n = 37)			30	21	18	11	8	14	27	27	11	17	2	9	7	5

^a^T1F, type 1 fimbriae.

**Figure 2:**
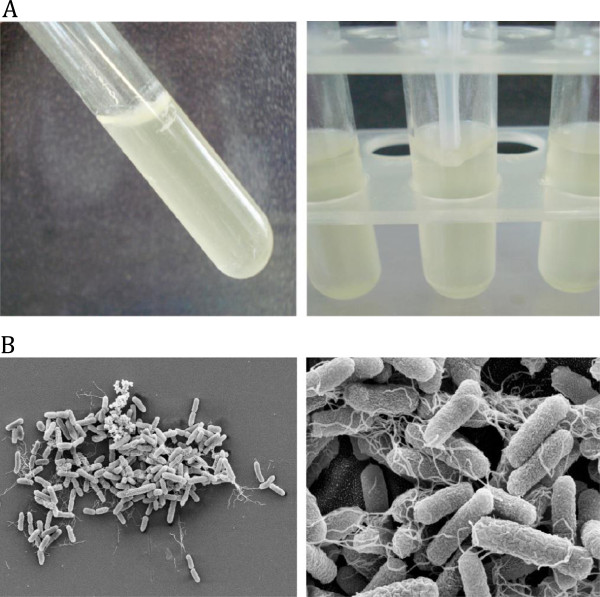
**Biofilm-formation ability of the A111 curli-negative strain. A** - Pellicle formation at the air-liquid interface; **B** - SEM micrographs of the biofilm formed on glass coverslips.

In attempt to identify genes potentially involved in biofilm formation, we selected one strong biofilm producer, but curli-negative, strain A111 of serotype O119:HND for mutagenesis. Like a previously described aEPEC O55:H7 strain, the A111 strain formed a strong biofim on an abiotic surface at 26°C, but not at 37°C, and a dense pellicle at the air-liquid interface (Figure 2). We generated a transposon library in the A111 strain using the EZ::TN < R6K*yori*/KAN-2 > Tnp transposome which confers resistance to kanamycin and screened the mutants for their capacity to produce biofilm. Among the 1,165-transposon mutants screened, eight biofilm deficient mutants were identified and compared to the wild type strain. All of them were deficient in their biofilm formation ability and did not form a pellicle at the air-liquid interface (data not shown). Identification of the transposon insertion sites was performed using PCR and sequence analysis. The DNA sequences of the mutants revealed similarity with type 1 fimbriae (three mutants; *fimC*, *fimD*, and *fimH*), diguanylate cyclase (one mutant, YP_006105849.1), ATP synthase F1, beta subunit (one mutant; atpD), and the uncharacterized YjiC protein (one mutant; YP_002394414.1). In two mutants the insertion site was too short for analysis.

This study showed that the ability to adhere to abiotic surfaces and form biofilm at low temperature is present in an array of aEPEC strains, and not restricted to a particular set of serotypes. In addition, our case control analysis showed an association between the biofilm-formation ability and diarrhea. These findings suggest that biofilm formation may play a role in the pathogenesis of some aEPEC strains.

A varied distribution of genes related to fimbrial and afimbrial adhesins reported in the literature to be associated with biofilm formation was identified among aEPEC strains. Recently, it has been shown that aEPEC strains harbor several pilus operons that could favor host colonization and environment survival [27]. In agreement with these findings, in our study, several adhesin genes were detected in most of the biofilm-producing strains. Interestingly, we observed an association between *fimA*/*fimH* genes and biofilm formation. Of the 37 biofilm producers, 27 (73%) carried the *fimA*/*fimH* genes versus 49 (55%) of 89 non-biofilm producers (P = 0.016). In addition, the curli genes, *csgA* and *crl* were found in 17 (46%) biofilm producers vs 28 (31%) non-biofilm producers (P = 0.041). No association was found between *fliC*, *flu*, *cah*, *ehaA*, or *hcpA* genes.

A recent study reported that the ability of an EPEC strain of serotype O55:H7 to produce biofilm at low temperature (26°C) was associated with curli [26]. In contrast, the A111 strain belonging to O119:HND formed a strong biofilm at 26°C pellicle at the air-liquid interface, but did not produce a red color on agar plates containing Congo red, implying that it does not produce curli. Three out of the six Tn*5* mutants analyzed had insertions in genes associated with type 1 fimbriae. It seems that, in this particular strain, type 1 fimbriae plays an important role in the interaction with abiotic surfaces, as the inactivation of the *fimC*, *fimD*, and *fimH* genes, significantly reduced its capacity to form biofilm and pellicle. In agreement with our findings, it has been recently reported that a mutation in the *fimA* gene of an aEPEC strain drastically affected its ability to form biofilm on plastic surface [28].

In addition, in one mutant, the inactivation of diguanylate cyclase also impaired biofilm formation. Cellular levels of c-di-GMP, a ubiquitous second messenger in bacteria, are controlled through the opposing activities of diguanylate cyclases and phosphodiesterases. Cyclic-di-GMP antagonistically controls motility and virulence of single, planktonic cells on one hand, and cell adhesion and persistence of multicellular communities on the other [29].

## Conclusion

This is the first study showing that the ability to adhere to abiotic surfaces and form biofilm is present in an array of aEPEC strains, and is not restricted to a particular set of serotypes. Our data suggest that type 1 fimbriae and diguanylate cyclase may be involved in aEPEC biofilm formation. Characterization of additional biofilm deficient mutants may reveal other mechanisms involved in biofilm formation and bring new insights into aEPEC pathogenesis.

## Methods

### Bacterial strains

The 126 aEPEC strains examined in this study belonged either to EPEC (*n* = 49) or non EPEC serotypes (*n* = 77), and were isolated during an epidemiological study of acute diarrhea in children under 2 years of age; the study was conducted in several cities of Brazil from 1999 through 2004 [30]. In the study, 1,102 stool specimens were obtained from children with diarrhea, presenting at the emergency room of public hospitals in seven cities representing different regions of Brazil, and 647 randomly selected children without any gastrointestinal symptoms from the same hospitals were studied. All strains were investigated for the presence of enteric pathogens such as diarrheagenic *E. coli*, *Shigella*, *Salmonella*, *Vibrio*, *Campylobacter*, *Giardia lamblia*, *Cryptosporidium,* and rotavirus. Atypical EPEC strains isolated as the only pathogen in stool samples were serotyped with specific antisera O1–O175 and H1–H56 acquired commercially (from the Universidad de Santiago de Compostela; Lugo, Spain). One isolate per subject was stored at -70°C in Luria Bertani broth (LB) with 15% glycerol.

### Ethics statement

The study was approved by the ethics committee of the Universidade Federal de São Paulo, Brazil. Stool samples were obtained with the written informed consent from the parents or guardians of the children with or without diarrhea.

### Biofilm formation assay

Biofilm assays were performed in triplicate using LB medium in 96-well polystyrene microtiter plates as previously described [26]. Briefly, strains were grown overnight at 37°C in LB. The optical density at 600 nm (OD_600_) of the culture was adjusted to 1.0 (corresponding to ca. 10^8^ CFU/ml). The cultures were then diluted 1:10 in fresh LB and used to inoculate three individual wells (200 μl per well). Plates were incubated statically at 26°C or 37°C for 24 h. Following these incubation periods, plates were washed three times with 250 μl/well of sterile phosphate-buffered saline (PBS). The plates were dried (26°C for 15 min), and each well was stained with 200 μl 0.1% crystal violet for 20 min. The OD_570_ values were determined in a microplate reader after solubilization of the dye with 95% ethanol (200 μl per well). The ability of aEPEC strains to form biofilm on abiotic surfaces was assessed by comparison with the standard biofilm producing enteroaggregative *E. coli* 042 strain (EAEC) and a non-biofilm forming strain (*E. coli* HB101). Strains were classified as biofilm producers when OD_570_ was higher than the *E. coli* HB101 strain mean value (OD_570_ ≤ 0.5). The biofilm producer strains were also subdivided into strong (OD_570_ ≥ 1.0) and weak (OD_570_ > 0.5 < 1) in comparison to the result of the EAEC 042 strain mean value (OD_570_ ≥ 1.0).

Biofilms were also analyzed by scanning electron microscopy (SEM). For SEM observations, coverslips in 24 well plates were used as adhesion surface. After incubation, the coverslips were washed three times with PBS and immersed in 2.5% glutaraldehyde in 0.1 M imidazole buffer (pH 7.0) for 2 h at room temperature. The preparations were then dehydrated in a graded series of ethanol solutions (50%, 80% and absolute), dried at critical point using CO_2_ as the transition fluid, and sputter-coated with gold.

### Pellicle formation

Strains were grown overnight in LB at 37°C and 5 ml was transferred into 4 ml LB in 15-ml glass tubes. After 48 h at 26°C or 37°C or the formation of biofilm (pellicle) at the air-liquid interface was visually observed and photographed with a digital camera.

### Detection of type 1 fimbriae and curli

Type 1 fimbriae expression was detected by the mannose-sensitive hemagglutination assay (MSHA) of guinea pig erythrocytes based on the method of Evans et al. [31] with some modifications. Bacterial suspensions (~3.0 × 10^8^ cells/ml) in PBS, prepared after growth in LB broth at 37°C for 18 h, were mixed with guinea pig red blood cells at room temperature in the presence or absence of 1% D-mannose. Curli production was evaluated on YESCA agar plates containing 40 mg/l of Congo Red (CR) and incubated for 24 h at 26°C or 37°C. Colony morphology was scored according to morphotypes previously described for *S. typhimurium*: red colonies are curli positive and white colonies, curli negative [32]. *E. coli* HB101 strain was used as a curli negative control.

### PCR assays

Strains were probed by PCR for the presence of *fimA* and *fimH* (type 1 fimbriae) [33,34], *fliC* (flagella) [35], *csgA* (curli structural subunit) [36], *hcpA (*hemorrhagic coli pilus) [37], *flu* (antigen 43) [38], cah (calcium-binding antigen 43 homologue) [39], and *ehaA* (Eha passenger domain) [40] genes.

### Transposon mutagenesis and genetic analysis

The aEPEC A111 strain was mutagenized with the EZ::TN < R6K*yori*/KAN-2 > Tnp transposome (Epicentre) according manufacturer’s instructions. Briefly, electrocompetent aEPEC cells were transformed with 1 μl of the Tnp transposome. Transposon-inserted bacterial colonies that grew on LB agar plates containing kanamycin were screened for biofilm formation as described above. Genomic DNA of the biofilm deficient mutants was digested with *Eco*RI and self-ligated, and used for transformation of *E. coli* DH5αλ*pir*. Rescued DNA plasmids were purified and sequenced using the transposon-specific primers R6KAN-2 RP-1 and KAN-2 FP-1 (Epicentre). DNA sequencing was performed at the Centro de Estudos do Genoma Humano-USP, São Paulo. Nucleotide sequence data were analyzed using SeqMan and MegAlign software and the BLAST tool (http://www.ncbi.nlm.nih.gov/BLAST).

### Nucleotide sequences and accession numbers

The DNA sequences for the six mutants are availability in NCBI database under accession numbers KM044265-KM044270, respectively.

## Competing interests

The authors declare that they have no competing interests.

## Authors’ contributions

HHN and LEPS performed experiments and analyzed data. RTS participated in the sequence alignment and sequence submission. NPS and ICAS wrote the manuscript. All authors read and approved the final manuscript.
